# The baseline predictive value of the RNA-binding proteins IGF2BP3/IMP3 and UPF1 for in-stent restenosis after percutaneous coronary intervention in acute coronary syndrome: a retrospective cohort study

**DOI:** 10.3389/fcvm.2026.2001481

**Published:** 2026-10-30

**Authors:** Yuying Liu, Haoran Wan, Bin Yang

**Affiliations:** Department of Cardiovascular Medicine, The Affiliated Hospital of Qingdao University, Qingdao, China

**Keywords:** in-stent restenosis (ISR), insulin-like growth factor II mRNA-binding protein 3 (IGF2BP3/IMP3), RNA-binding protein, up-frameshift 1 (UPF1), vascular endothelial growth factor (VEGF)

## Abstract

**Objective:**

To investigate the predictive capacity of baseline (pre-procedural) RNA-binding proteins insulin-like growth factor II mRNA-binding protein 3 (IGF2BP3/IMP3), upstream frameshift protein 1 (UPF1), and in-stent restenosis (ISR) after percutaneous coronary intervention (PCI) in patients with acute coronary syndrome (ACS).

**Methods:**

A total of 1,380 ACS patients who underwent PCI at the Affiliated Hospital of Qingdao University from February 2020 to February 2024 were divided into ISR (*n* = 216) and non-ISR (*n* = 1,164) groups based on coronary angiography results 6 months post-procedure. Clinical, laboratory, and echocardiographic data were compared. Multivariate logistic regression identified ISR risk factors, and receiver operating characteristic (ROC) analysis evaluated the predictive value of IGF2BP3/IMP3 and UPF1.

**Results:**

VEGF and IGF2BP3/IMP3 levels were significantly lower, while UPF1 was higher, in the ISR group (*P* < 0.05). Compared with the non-ISR group, patients in the ISR group were significantly older, had higher proportions of current smokers and diabetes mellitus, elevated CRP levels, and worse echocardiographic parameters (*P* < 0.05). Average stent length, stent numbers, and SYNTAX scores were also significantly different between the two groups (*P* < 0.05). There were no significant differences in male gender, hypertension prevalence, or culprit vessel distribution. Multivariate logistic regression adjusted for clinical covariates (age, sex, current smoking, hypertension, and diabetes mellitus) identified that IGF2BP3/IMP3 (adjusted OR = 0.551, 95% CI: 0.354–0.860, *P* = 0.009) was independently associated with lower odds of ISR, whereas UPF1 (adjusted OR = 5.496, 95% CI: 1.906–15.852, *P* = 0.002) was independently associated with an increased likelihood of ISR (*P* < 0.05). Furthermore, IGF2BP3/IMP3 was positively correlated with VEGF (*r* = 0.192, *P* < 0.001), whereas UPF1 was negatively correlated with VEGF (*r* = −0.239, *P* < 0.001). The UPF1/IGF2BP3/IMP3 ratio had the highest statistical predictive value for ISR (AUC = 0.870, 95% CI: 0.846−0.894, optimism-corrected AUC = 0.870, sensitivity 79.6%, specificity 79.0%).

**Conclusion:**

Decreased IGF2BP3/IMP3 and VEGF and increased UPF1 are associated with ISR after PCI. The baseline UPF1/(IGF2BP3/IMP3) ratio provides strong longitudinal predictive value for post-PCI ISR in ACS patients.

## 1 Introduction

Percutaneous coronary intervention (PCI) is currently the predominant strategy for the treatment of coronary heart disease, which is explored to alleviate myocardial ischemia through mechanically widening the narrowed or blocked arterial segments (1). The application of bare metal stents (BMS) in most myocardial revascularization procedures can effectively reverse acute vascular occlusion while reducing the risk of coronary complications. Nevertheless, coronary stent implantation is continually plagued by neointimal hyperplasia and postoperative intrastent restenosis, which is the result of excessive vascular healing due to stent-related injury (2). To compensate for the defects and limitations of BMS, drug-eluting stents (DES) have been designed to safely and effectively reduce restenosis in the recent years (3). DES are usually encapsulated with cytotoxic drugs or cell suppressor to prevent vascular smooth muscle cell (VSMC) proliferation and intimal hyperplasia, thus minimizing the risk of in-stent restenosis (ISR) (4, 5). Unfortunately, DES can also impair the normal healing response that result in an increased risk of late-onset and extremely late-onset stent thrombosis owing to the non-selective characteristic of these drugs (6–9). Stent thrombosis has become an prominent rationale of recurrent angina pectoris, myocardial infarction and even sudden death in patients with stent implantation. Therefore, it is a pressing issue that requires addressing to reconcile the vascular healing response with thrombosis for decreasing ISR.

Despite advances in contemporary DES, ISR remains a clinically relevant complication after PCI. Its pathogenesis is multifactorial, involving mechanical factors such as stent underexpansion and fracture, as well as biological processes including neointimal hyperplasia and neoatherosclerosis. Modern ISR management emphasizes employing intravascular imaging to identify underlying mechanisms, thereby guiding individualized treatment strategies (10, 11).

Unequivocally speaking, the proliferation and migratory competence of endothelial cells is crucial in the process of vascular reendothelialization and plays an imperative role within ISR (12). Previous studies have implicated that the delay of reendothelialization on the stent surface is critical to the occurrence of ISR (13). In other words, the activation of reendothelialization after arterial injury attenuates the formation of neointima, which is considered a new therapeutic strategy to improve endothelial recovery after PCI procedure-induced vascular injury. Vascular endothelial growth factor (VEGF) is a pivotal pro-angiogenic factor that promotes reendothelialization due to its unique regulation of endothelial cells (14–16). A recent study has indicated a significant correlation between VEGF and Insulin-like growth factor II mRNA-binding protein 3 (IGF2BP3/IMP3) in lung adenocarcinoma (17). IGF2BP3/IMP3 is a highly conserved family of single-stranded RNA-binding proteins that play cardinal roles in cell metabolism, adhesion, and differentiation. Somasundaram *et al*. also demonstrated that IGF2BP3/IMP3 facilitated glioblastoma growth, angiogenesis, and tumor metastasis (18). Additionally, Yan et al. have attested that IGF2BP3/IMP3 plays an essential role in promoting VEGF mRNA stabilization, which consequently enhances endothelial cell proliferation and migration (19). Upstream frameshift protein (UPF1) is also an RNA-binding protein for VEGF in addition to IGF2BP3/IMP3, which plays an integral role in VEGF regulation. UPF1 is reported as a pivotal factor in nonsense-mediated decay (NMD), which is an mRNA decay pathway engaged in RNA quality control and fine-tuning of gene expression (20–23). Specifically, UPF1 eradicates anomalous mRNAs containing premature termination codons and modulates the homeostatic levels of standard physiological mRNAs (24). Combined with previous studies, it was substantiated that the RNA-binding proteins IGF2BP3/IMP3 and UPF1 could regulate the expression of VEGF. It is speculated that IGF2BP3/IMP3 and UPF1 may accelerate the reendothelialization of injured vessels through regulating the expression of VEGF. To this date, the specific variations of the RNA-binding protein IGF2BP3/IMP3 and UPF1 in the process of reendothelialization in patients with ISR after PCI and their relevance remains obscure. Therefore, the objective of this study is to investigate the association between baseline serum IGF2BP3/IMP3 and UPF1 levels in patients with acute coronary syndrome (ACS) prior to PCI and the risk of ISR occurrence at 6 months post-operation, as well as their predictive value for such adverse event.

## 2 Materials and methods

### 2.1 Ethical approval

This study was conducted in accordance with the principles of the Declaration of Helsinki and was approved by the Medical Ethics Committee of the Affiliated Hospital of Qingdao University (IRB approval number: QYFY WZLL 28688). Due to the retrospective nature of the study and the use of anonymized patient data, the requirement for written informed consent was waived by the ethics committee.

### 2.2 Study population

During the study period from February 2020 to February 2024, a total of 1,748 ACS patients who underwent PCI were consecutively screened. All patients were routinely recommended to undergo scheduled follow-up coronary angiography at 6 months post-procedure according to our institutional surveillance protocol, or earlier if recurrent ischemic symptoms occurred.

After applying exclusion criteria, 106 patients were excluded (severe hepatic/renal dysfunction: *n* = 28; infection: *n* = 22; malignant tumors: *n* = 25; connective tissue disease: *n* = 11; antiplatelet resistance/non-adherence: *n* = 20). Among 1,642 eligible patients, 262 patients (16.0%) did not undergo invasive angiographic follow-up at 6 months (189 refused invasive angiography, 48 were lost to contact, and 25 underwent coronary CTA only). Consequently, a final cohort of 1,380 patients with confirmed 6-month angiographic outcomes was enrolled (Figure 1).

**Figure 1:**
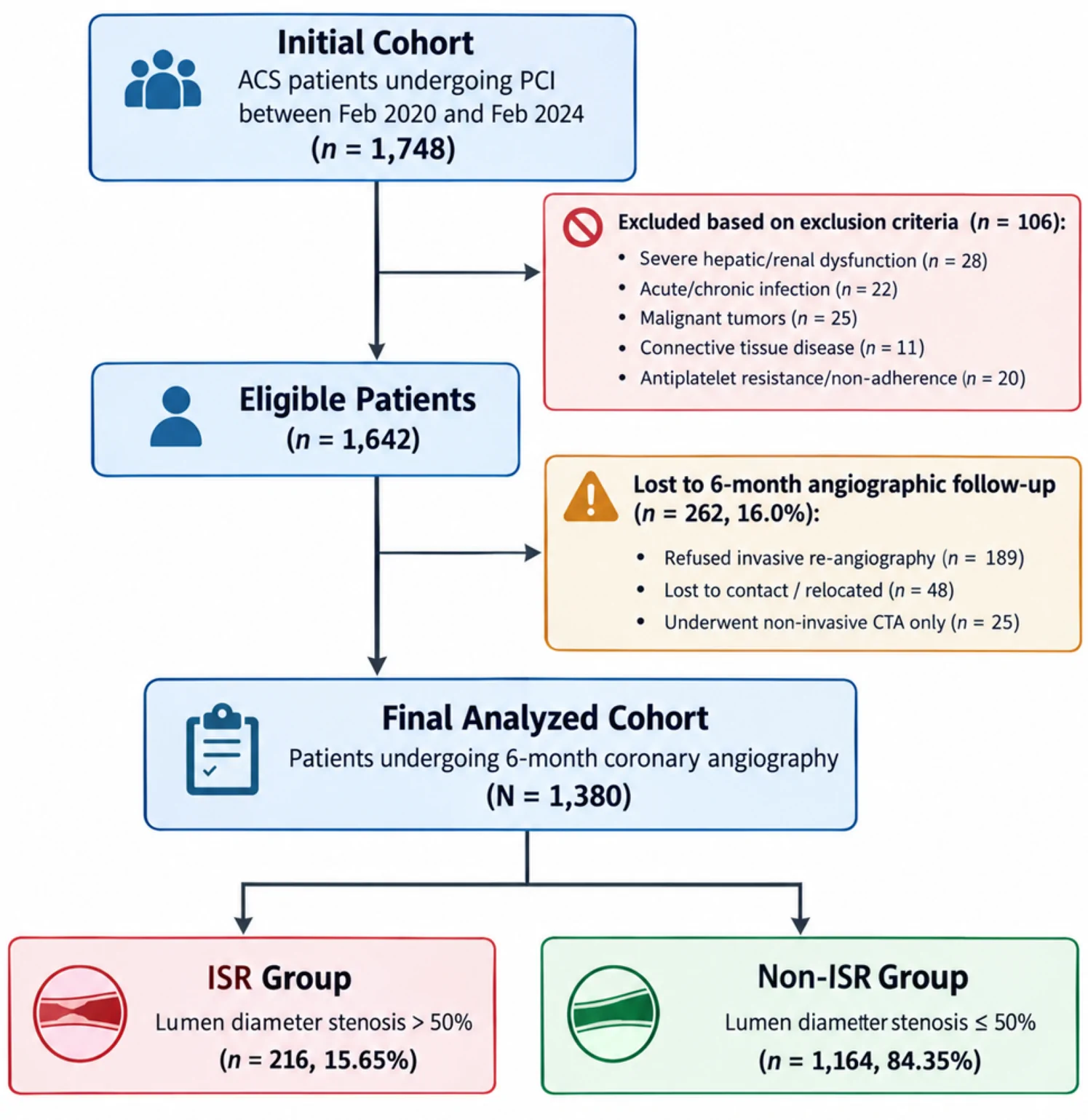
Flowchart of patient enrollment and study population selection.

The primary endpoint of this study was angiographic ISR, defined as a binary outcome of greater than 50% diameter stenosis within the stent or within 5 mm of either stent edge at the 6-month angiographic follow-up, irrespective of whether target lesion revascularization (TLR) was clinically performed. Angiographic assessment was conducted locally at our cardiovascular catheterization center utilizing an offline Quantitative Coronary Angiography (QCA) system. All angiographic films were independently evaluated by two experienced interventional cardiologists who were strictly blinded to the patients' clinical data, baseline characteristics, and laboratory biomarker measurements (VEGF, IGF2BP3/IMP3, and UPF1 levels). Discrepancies between the two observers were resolved by consensus or adjudication by a third senior interventional cardiologist. Inter-observer reproducibility showed substantial agreement (Cohen's *κ* = 0.89). Enrolled patients were ultimately categorized into the ISR group (*n* = 216) and the non-ISR group (*n* = 1,164). Selection criteria: 1) fulfillment of ACS diagnostic criteria; 2) pharmacological treatment for official secondary prevention of coronary artery disease after PCI. Exclusion criteria: 1) aspirin and clopidogrel resistance; 2) severe hepatic and renal insufficiency; 3) acute and chronic infection; 4) malignant tumor; and 5) connective tissue disease. All enrolled patients received second-generation drug-eluting stents (DES); bare-metal stents (BMS) were not used in this cohort. Standard guideline-directed dual antiplatelet therapy (DAPT; aspirin 100 mg/day plus clopidogrel 75 mg/day or ticagrelor 90 mg twice daily) was administered to all patients for a minimum of 12 months post-PCI. Age, sex, history of smoking, history of hypertension, history of diabetes mellitus, biochemical indices, echocardiographic examination results and fundamental stenting were well documented in the ISR and non-ISR groups.

### 2.3 Biochemical index detection

After admission, venous blood samples were collected from both groups, followed by the detection of total cholesterol, triglycerides, low-density lipoprotein cholesterol, high-density lipoprotein cholesterol, fasting blood glucose and C-reactive protein.

### 2.4 Measurement of cardiac ultrasound quantitative data and stent implantation

Cardiac echocardiographic results were gathered from both groups adopting a GE Vivid E95 ultrasound system (GE Healthcare, Chicago, IL, USA), and left ventricular ejection fraction, septal thickness, number of stenting procedures, and stent length were documented.

### 2.5 Determination of serum VEGF, IGF2BP3/IMP3 and UPF1

Venous blood samples (3–5 mL) were collected upon admission (baseline, prior to PCI procedure) as well as on day 3, day 7 and day 14 post-PCI. Baseline samples collected at admission were specifically utilized for the primary comparative analysis, multivariable logistic regression modeling, and ROC curve evaluations. All enrolled 1,380 patients had complete baseline biomarker measurements without missing data. Due to routine outpatient follow-up attrition, serial blood samples post-PCI were available for 1,332 patients on Day 3, 1,245 patients on Day 7, and 1,145 patients on Day 14. Post-PCI day 3, day 7 and day 14 samples were employed exclusively to evaluate the dynamic temporal alterations of these biomarkers during the early vascular response. After collection, blood samples were centrifuged at 2,500 rpm for 20 min at 4 °C. The supernatant (serum) was aliquoted and stored at −80 °C (with initial temporary placement at −20 °C for < 24 h) to prevent protein degradation and repeated freeze-thaw cycles until batch testing under identical conditions. Serum levels of IGF2BP3/IMP3, UPF1, and VEGF were quantified using commercial sandwich enzyme-linked immunosorbent assay (ELISA) kits purchased from MyBioSource (San Diego, CA, USA) strictly according to the manufacturer's instructions: Human VEGF ELISA Kit (Cat. No. MBS355343; detection range: 31.2–2,000 pg/mL, sensitivity: < 1 pg/mL), Human IGF2BP3/IMP3 ELISA Kit (Cat. No. MBS2505670; detection range: 0.313–20 ng/mL pg/mL, sensitivity: Min: 0.188 ng/mL; Max: 20 ng/mL), and Human UPF1 ELISA Kit (Cat. No. MBS9320003; detection range: 62.5 −2,000 pg/mL, sensitivity: < 10pg/mL). All serum samples were tested in duplicate without dilution. Standard curves were calibrated using four-parameter logistic (4-PL) curve fitting. Both intra-assay and inter-assay coefficients of variation (CV) across all runs were < 15% and < 15%, respectively. Laboratory technicians performing the assays were strictly blinded to patient identities, clinical data, and 6-month angiographic outcomes.

### 2.6 Statistical analysis

All analyzed 1,380 patients had complete records for baseline clinical, laboratory, and procedural variables; therefore, a complete-case analysis was conducted without the need for data imputation. Statistical analysis was calculated utilizing SPSS 27.0 statistical software. The normality of continuous variables was assessed employing the Shapiro–Wilk test alongside visual inspection of normal Q-Q plots. Continuous variables with a normal distribution (including age, stent length, stent number, LVEF, IVS, TC, LDL-C, as well as serum levels of VEGF, IGF2BP3/IMP3, and UPF1) are presented as mean ± standard deviation (SD) and compared between groups using the independent-samples Student's t-test (or Welch's *t*-test for unequal variances). Non-normally distributed (skewed) continuous variables (such as CRP, TG, and SYNTAX score) are expressed as median (interquartile range, P25–P75) and compared using the Mann–Whitney U test. Qualitative variables are described as frequencies and percentages (n, %), and compared using the Pearson chi-square test or Fisher's exact test. To evaluate the relationship between circulating biomarkers, both Spearman rank correlation (primary, due to the non-normal distribution of UPF1) and Pearson linear correlation (with 95% confidence intervals [CIs]) were performed. Linear regression trendlines with 95% confidence bands were fitted to the scatterplots. Qualitative information was described as the number of cases (%), and comparisons between the two groups were drawn employing the X2 test or Fisher's exact probability method. Multiple logistic regression was methodically implemented to evaluate the independent influencing factors of ISR utilizing baseline biomarker levels. To control for confounding, candidate covariates were selected based on established clinical risk factors for ISR and baseline demographic differences, including age, sex, current smoking status, hypertension, and diabetes mellitus. With 216 ISR events and 8 independent variables in the multivariable model, the events per variable (EPV) ratio was 27, well above the recommended threshold of 10 to minimize overfitting.

Multicollinearity was formally assessed exploiting variance inflation factors (VIF) and tolerance statistics, where VIF < 5 was considered acceptable. Model goodness-of-fit and calibration were evaluated using the Hosmer–Lemeshow test (with *P* > 0.05 indicating adequate fit), and model discrimination was determined by the concordance index (C-statistic/AUC). To evaluate dynamic temporal changes of serum biomarkers across the four repeated time points (baseline, Day 3, Day 7, and Day 14), one-way repeated-measures analysis of variance (ANOVA) with Greenhouse–Geisser correction was performed to account for within-subject correlation, followed by Bonferroni *post-hoc* tests for pairwise comparisons. Receiver operating characteristic (ROC) curves were constructed to determine discriminatory ability. The biological rationale for constructing the UPF1/IGF2BP3/IMP3 ratio was prespecified based on their established opposing functions in regulating VEGF mRNA stability and vascular healing. The algebraic quotient (UPF1/IGF2BP3/IMP3) was calculated using baseline raw concentrations to facilitate clinical interpretability. Sensitivity analyses using log-transformed variables [ln(UPF1)−ln(IGF2BP3/IMP3)] yielded identical discriminatory accuracy. The optimal cut-off point of baseline UPF1/IGF2BP3/IMP3 was identified using the maximum Youden index. To mitigate potential optimism and overfitting, internal validation was performed utilizing 1,000 bootstrap resamples to calculate the optimism-corrected AUC. Sensitivity, specificity, positive predictive value (PPV), and negative predictive value (NPV) along with their respective 95% confidence intervals were reported. Pairwise comparisons of ROC curves (UPF1/(IGF2BP3/IMP3) vs. IGF2BP3/IMP3 alone, UPF1 alone, and baseline clinical risk models) were formally evaluated using the DeLong test. The incremental predictive value of UPF1/IGF2BP3/IMP3 beyond traditional clinical covariates was assessed employing the Concordance index (C-index), continuous Net Reclassification Improvement (NRI), and Integrated Discrimination Improvement (IDI). The difference was statistically significant (*P* < 0.05).

## 3 Results

### 3.1 Comparison of clinical data between the two groups of patients

Baseline demographic, clinical, laboratory, and procedural characteristics of the study population are summarized in Table 1. Patients in the ISR group were significantly older (69.75 ± 8.51 vs. 66.35 ± 8.95 years, *P* < 0.001) and exhibited significantly higher proportions of current smokers (37.96% vs. 27.32%, *P* = 0.002) and diabetes mellitus (14.35% vs. 5.93%, *P* < 0.001) compared with the non-ISR group. In addition, serum CRP, TC, TG, and HDL-C levels exhibited statistically significant differences between the two groups (*P* ≤ 0.001). Regarding angiographic and echocardiographic findings, patients with ISR had significantly higher stent numbers (1.98 ± 0.40 vs. 1.93 ± 0.25, *P* = 0.036), shorter average stent length (32.35 ± 0.38 vs. 33.55 ± 0.44 mm, *P* < 0.001), higher SYNTAX scores (*P* = 0.048), lower LVEF (56.65 ± 9.31% vs. 60.90 ± 8.95%, *P* < 0.001), and thicker IVS (1.23 ± 0.31 vs. 1.16 ± 0.20 cm, *P* < 0.001). No statistically significant differences were observed regarding male gender, hypertension history, ACS classification, or culprit vessel distribution (*P* > 0.05).

**Table 1:** Comparison of clinical data between the two groups of patients.

Variables	ISR (*n* = 216)	Non-ISR(*n* = 1,164)	*P* value
age, years	69.75 ± 8.51	66.35 ± 8.95	< 0.001
Male gender, *n* (%)	123 (56.94)	657 (56.44)	0.951
Current smoker, *n* (%)	82 (37.96)	318 (27.32)	0.002
Hypertension, *n* (%)	175 (81.02)	875 (75.17)	0.078
Diabetes mellitus, *n* (%)	31 (14.35)	69 (5.93)	< 0.001
CRP (mg•L^−1^)	0.95 (0.36, 3.04)	0.45 (0.27, 0.73)	< 0.001
TC (mmol•L^−1^)	3.63 ± 1.40	4.45 ± 1.11	< 0.001
TG (mmol•L^−1^)	1.14 (0.67,1.76)	1.24 (0.97,1.71)	0.001
LDL-C(mmol•L^−1^)	2.68 ± 1.03	2.69 ± 0.98	0.852
HDL-C(mmol•L^−1^)	1.13 ± 0.24	1.05 ± 0.26	< 0.001
ACS classification			0.481
UA, *n* (%)	132 (61.11)	675 (57.99)	
NSTEMI, *n* (%)	54 (25.00)	338 (29.04)	
STEMI, *n* (%)	30 (13.89)	151 (12.97)	
Stent numbers, n	1.98 ± 0.40	1.93 ± 0.25	0.036
Average stent length (mm)	32.35 ± 0.38	33.55 ± 0.44	< 0.001
SYNTAX score	12.82 (7.83, 19.83)	11.82 (8.74, 15.83)	0.048
Culprit vessel			0.618
LAD, *n* (%)	87 (40.28)	473 (40.64)	
LCX, *n* (%)	76 (35.19)	375 (32.22)	
RCA, *n* (%)	53 (24.54)	316 (27.15)	
LVEF (%)	56.65 ± 9.31	60.90 ± 8.95	< 0.001
IVS (cm)	1.23 ± 0.31	1.16 ± 0.20	< 0.001

Values are presented as mean ± SD for normally distributed continuous variables, median (interquartile range, P25–P75) for skewed continuous variables, and number (percentage, %) for categorical variables. Between-group comparisons were evaluated using the independent-samples Student's t-test for normally distributed continuous variables, the Mann–Whitney U test for skewed continuous variables (CRP, TG, and SYNTAX score), and the Pearson Chi-square test (or Fisher's exact test) for categorical variables.

ISR: in-stent restenosis; CRP: C-reactive protein; TC: total cholesterol; TG: triglyceride; LDL-C: Low density lipoprotein cholesterol; HDL-C: High density lipoprotein cholesterol; ACS: acute coronary syndrome; UA: unstable angina; NSTEMI: Non-ST elevation myocardial infarction; STEMI: ST elevation myocardial infarction; LAD: left anterior descending; LCX: left circumflex coronary artery; RCA: right coronary artery; LVEF: Left ventricular ejection fraction; IVS: Interventricular septum thickness.

### 3.2 Comparison of VEGF, IGF2BP3/IMP3 and UPF1 between the two groups of patients

Baseline serum VEGF and IGF2BP3/IMP3 levels (measured upon admission prior to PCI) were significantly lower in the ISR group than in the non-ISR group (all *P* < 0.001). While baseline serum UPF1 levels were considerably increased in the ISR group compared with the non-ISR group (*P* < 0.001) (Table 2).

**Table 2:** Comparison of baseline VEGF, IGF2BP3/IMP3 and UPF1 between the two groups of patients.

Variables	ISR (*n* = 216)	Non-ISR (*n* = 1,164)	*P* value
VEGF (pg•mL^−1^)	128.57 ± 13.60	136.25 ± 12.50	< 0.001
IGF2BP3/IMP3(pg•mL^−1^)	25.36 ± 3.43	40.11 ± 8.29	< 0.001
UPF1(pg•mL^−1^)	63.19 ± 5.24	44.93 ± 2.62	< 0.001

Data are presented as mean ± standard deviation (SD). Between-group comparisons were performed using Welch's independent-samples t-test.

ISR, in-stent restenosis; VEGF, vascular endothelial growth factor; IGF2BP3/IMP3, insulin-like growth factor II mRNA-binding protein 3; UPF1, UP-frameshift 1.

### 3.3 Multivariable logistic regression analysis of risk factors for ISR

Multivariable logistic regression was performed to identify the risk factors for ISR, taking in-stent restenosis (ISR = 1, Non-ISR = 0) as the dependent variable and baseline IGF2BP3/IMP3, UPF1, and VEGF (along with clinically significant covariates) as dependent variable and IGF2BP3/IMP3, UPF1, and VEGF as independent variables, adjusting for established clinical covariates including age, sex, current smoking, hypertension, and diabetes mellitus. The collinearity diagnosis exhibited that there was no significant multicollinearity among the variables. The Hosmer-Lemeshow test indicated that the fit and calibration were good, and the overall discriminative ability of the multivariate model was strong.

The results revealed that VEGF (adjusted OR = 1.051, 95% CI: 0.859–1.286, *P* = 0.626) was not an independent predictor, whereas IGF2BP3/IMP3 (adjusted OR = 0.551, 95% CI: 0.354–0.860, *P* = 0.009) was an independent variable inversely associated with ISR occurrence, and UPF1 (adjusted OR = 5.496, 95% CI: 1.906–15.852, *P* = 0.002) was an independent statistical risk predictor for the development of ISR (Table 3).

**Table 3:** Multivariate logistic regression analysis of risk factors for ISR.

Variables	OR	95%CI	*P* value
VEGF	1.051	0.859–1.286	0.626
IGF2BP3/IMP3	0.551	0.354–0.860	0.009
UPF1	5.496	1.906–15.852	0.002

ORs were adjusted for age, sex, current smoking, hypertension, diabetes mellitus. Events per variable (EPV) = 27. All variance inflation factors (VIF) were < 2.0.

OR, odds ratio; CI, confidence interval; VEGF, vascular endothelial growth factor; IGF2BP3/IMP3, Insulin like growth factor II mRNA-binding protein 3(IGF2BP3/IMP3); UPF1, Up-frameshift 1.

### 3.4 Correlation analysis of serum IGF2BP3/IMP3, UPF1 and VEGF

Correlation analysis demonstrated that baseline serum IGF2BP3/IMP3 was positively correlated with VEGF (Pearson *r* = 0.192, 95% CI: 0.141–0.242, *P* < 0.001; Spearman rho=0.185, *P* < 0.001; Figure 2). In contrast, serum UPF1 showed an inverse correlation with VEGF (Pearson *r* = −0.239, 95% CI: −0.288 to −0.189, *P* < 0.001; Spearman rho = −0.170, *P* < 0.001; Figure 3).

**Figure 2:**
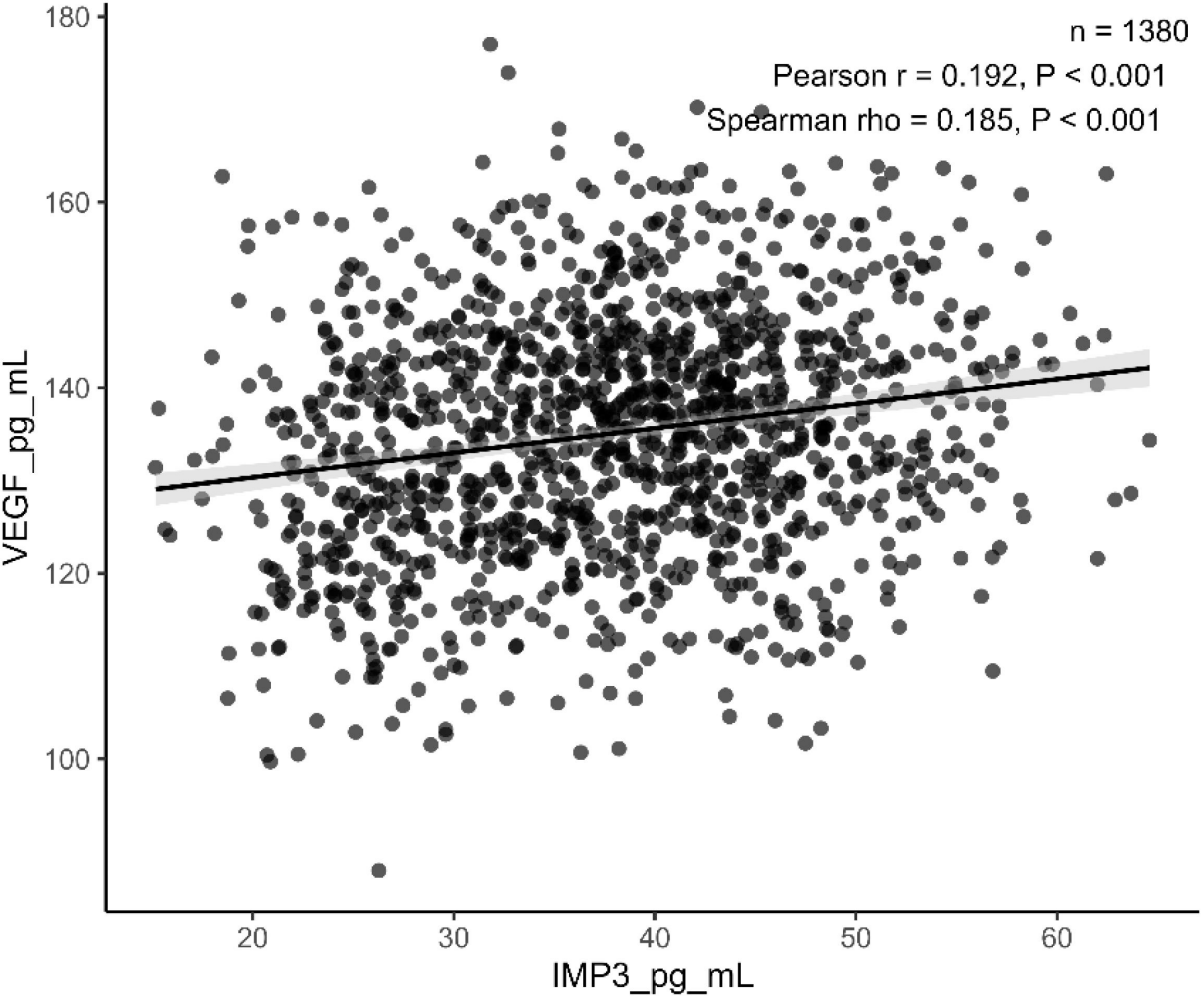
Correlation analysis of VEGF and IGF2BP3/IMP3.

**Figure 3:**
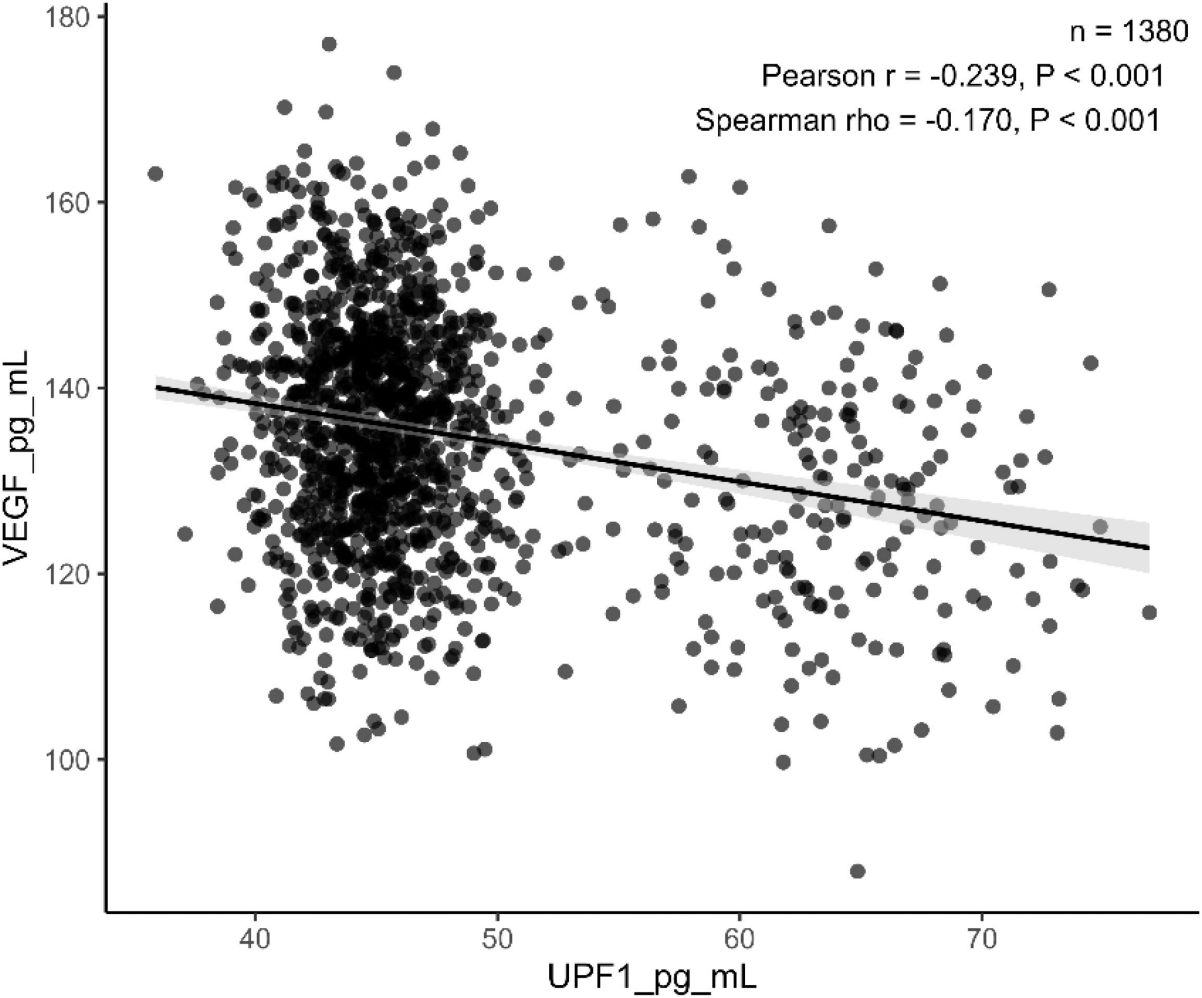
Correlation analysis of VEGF and UPF1.

### 3.5 Temporal variation of IGF2BP3/IMP3, UPF1, and VEGF after PCI

To further investigate the dynamic changes of the studied molecules during the reendothelialization progression, we analyzed their serum levels at four consecutive time points (baseline: *n* = 1,380; day 3: *n* = 1,132; day 7: *n* = 1,245; and day 14: *n* = 1,145 post-PCI). One-way repeated-measures ANOVA revealed significant temporal alterations across time for IGF2BP3/IMP3 (F = 9,008.01, *P* < 0.001, *η*^2^_G_ = 0.223), VEGF (F = 7,011.04, *P* < 0.001, *η*^2^_G_ = 0.191), and UPF1 (F = 5,622.62, *P* < 0.001, *η*^2^_G_ = 0.207).

As shown in Figure 4, serum levels of IGF2BP3/IMP3 and VEGF began to rise on day 3 post-PCI, reached their peak levels on day 7, and subsequently maintained a sustained high concentration through day 14. In contrast, the serum level of UPF1 showed an opposite trend, starting to decrease on day 3, reaching its nadir on day 7, and then remained stable at a low plateau without significant difference between day 7 and day 14 (Bonferroni-adjusted *P* = 1.00).

**Figure 4:**
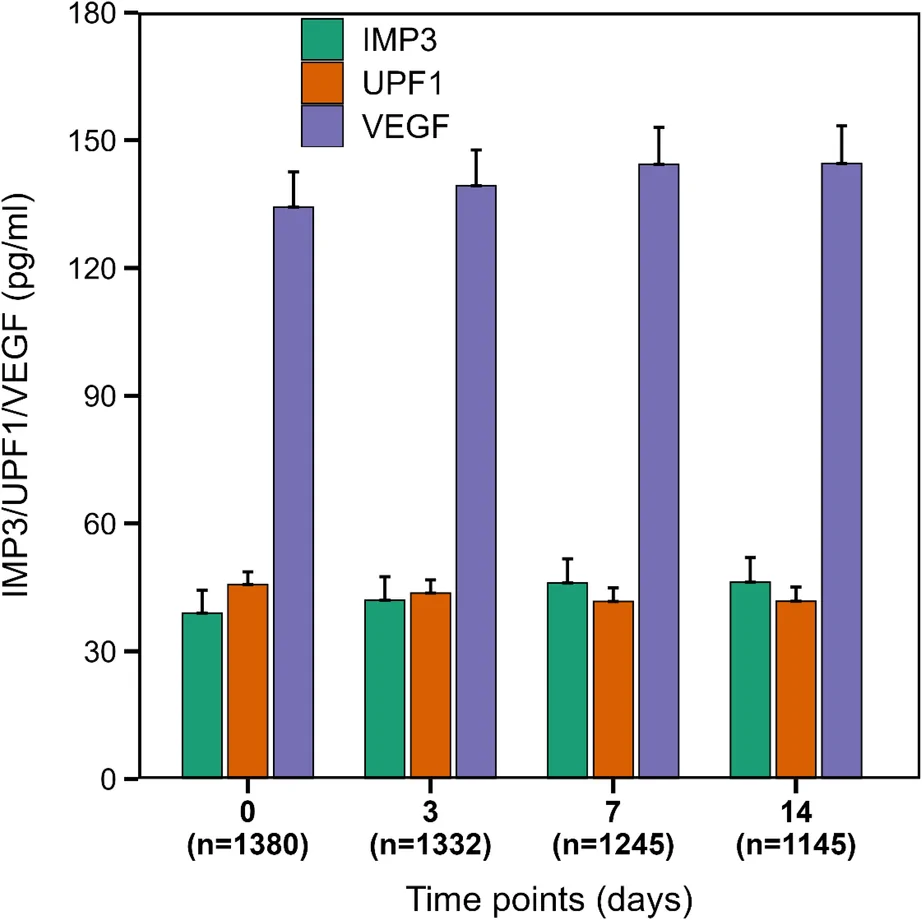
Temporal variation of serum IGF2BP3/IMP3, UPF1, and VEGF levels after PCI across baseline, day 3 (*n* = 1,332), day 7 (*n* = 1,245), and day 14 (*n* = 1,145). Data are presented as mean ± standard deviation (SD). Longitudinal changes across time points were analyzed using one-way repeated-measures ANOVA (all *P* < 0.001).

### 3.6 Predictive value and internal validation of UPF1 and IGF2BP3/IMP3

Based on the maximum Youden index, the optimal cut-off value of the baseline UPF1/(IGF2BP3/IMP3) ratio for predicting ISR was 1.30. Internal validation using 1,000 bootstrap resamples yielded an optimism-corrected AUC of 0.870 (apparent AUC = 0.870, 95% CI: 0.846–0.894, *P* < 0.001). At this cut-off, 416 patients were classified above the threshold (172 ISR and 244 non-ISR) and 964 patients below the threshold (44 ISR and 920 non-ISR). The baseline UPF1/IGF2BP3/IMP3 ratio exhibited a sensitivity of 79.6% (95% CI: 73.6%–84.8%), specificity of 79.0% (95% CI: 76.6%–81.3%), positive predictive value (PPV) of 41.3% (95% CI: 36.6%–46.2%), and negative predictive value (NPV) of 95.4% (95% CI: 93.9%–96.7%) (Figure 5, Table 4).

**Figure 5:**
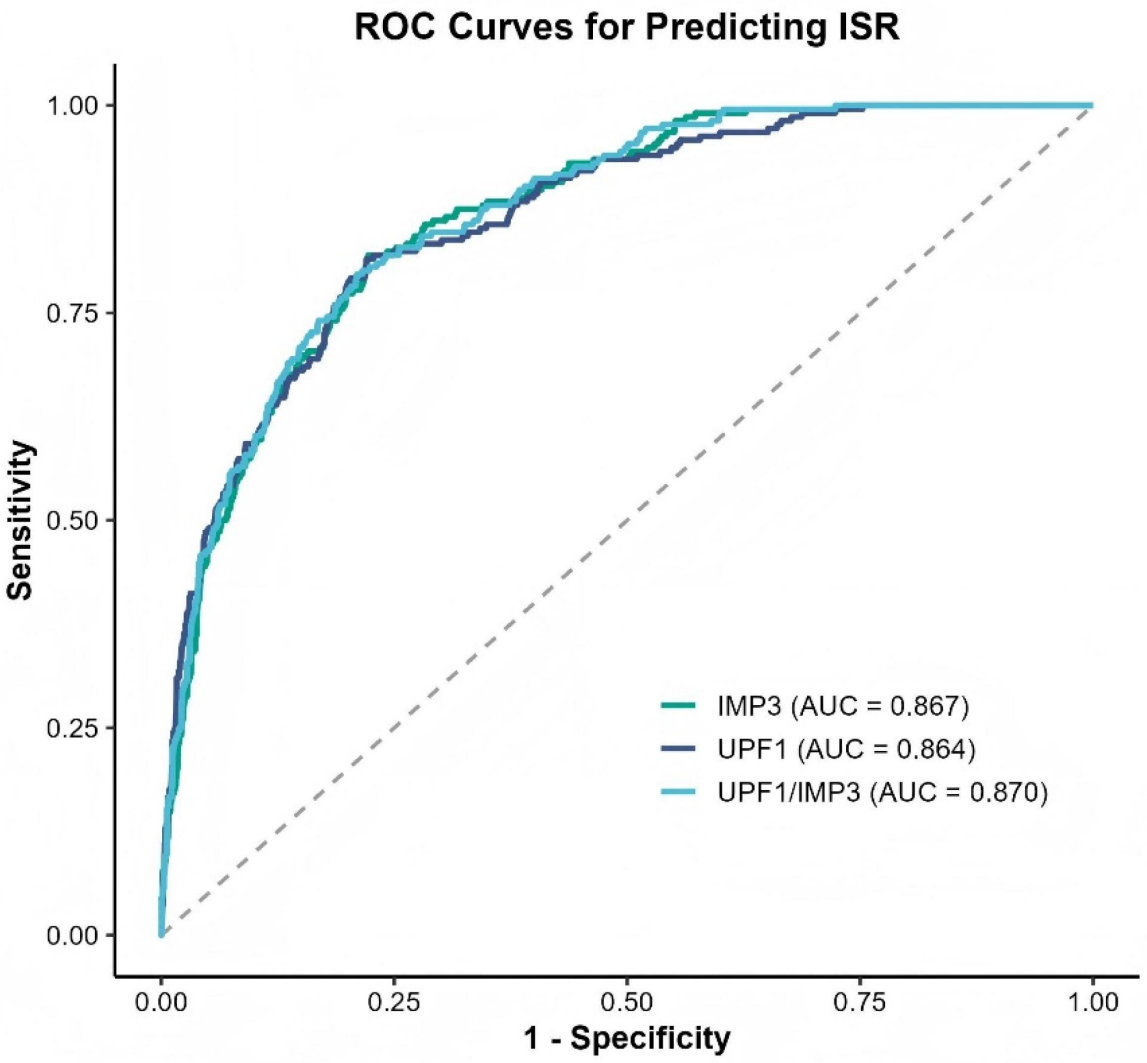
ROC curves of IGF2BP3/IMP3, UPF1, and the UPF1/(IGF2BP3/IMP3) ratio for predicting ISR.

**Table 4:** ROC curve analysis.

Variables	IGF2BP3/IMP3	UPF1	UPF1/IGF2BP3/IMP3
Cutoff	36.61	47.07	1.30
AUC (95% CI)	0.867 (0.844–0.891)	0.864 (0.838–0.889)	0.870 (0.846–0.894)
Optimism-corrected AUC	0.867	0.863	0.870
Sensitivity (95% CI)	81.9% (76.2–86.8%)	81.5% (75.6–86.4%)	79.6% (73.6–84.8%)
Specificity (95% CI)	77.8% (75.3–80.2%)	78.0% (75.5–80.4%)	79.0% (76.6–81.3%)
PPV (95% CI)	40.7% (36.0–45.5%)	40.7% (36.1–45.5%)	41.3% (36.6–46.2%)
NPV (95% CI)	95.9% (94.4–97.0%)	95.8% (94.3–97.0%)	95.4% (93.9–96.7%)
*P* value	< 0.001	< 0.001	< 0.001
DeLong *P* vs. UPF1/IGF2BP3/IMP3	0.242	0.152	Reference

All analyses were performed utilizing baseline serum measurements obtained prior to PCI. IGF2BP3/IMP3: Insulin like growth factor II mRNA-binding protein 3(IGF2BP3/IMP3); UPF1: Up-frameshift 1. Sensitivity analysis using ln(UPF1)−ln(IGF2BP3/IMP3) showed an identical AUC of 0.870 (95% CI: 0.846–0.894). Addition of the baseline UPF1/IGF2BP3/IMP3 ratio to the clinical risk model (age, sex, smoking, hypertension, diabetes, stent length, and SYNTAX score) significantly improved the AUC from 0.982 (95% CI: 0.974–0.990) to 0.991 (95% CI: 0.986–0.996; DeLong *P* < 0.001; AIC dropped from 338.86 to 242.08; continuous NRI = 1.097, 95% CI: 0.795–1.454, *P* < 0.001; IDI = 0.078, 95% CI: 0.051–0.115, *P* < 0.001).

Sensitivity analysis utilizing natural log-transformed values [ln(UPF1)−ln(IGF2BP3/IMP3)] demonstrated an identical AUC of 0.870 (95% CI: 0.846–0.894, sensitivity 79.6%, specificity 79.0%), confirming that biomarker skewness did not distort model performance. Pairwise DeLong tests indicated that the standalone AUC of the UPF1/(IGF2BP3/IMP3) ratio (0.870) did not reach statistical difference compared to IGF2BP3/IMP3 alone (0.867, *P* = 0.242) or UPF1 alone (0.864, *P* = 0.152). However, the baseline clinical model incorporating the UPF1/(IGF2BP3/IMP3) ratio markedly improved model fitness（AIC decreased from 338.86 to 242.08）and enhanced discrimination, elevating the AUC from 0.982 (95% CI: 0.974–0.990) to 0.991 (95% CI: 0.986–0.996, DeLong *P* < 0.001). Furthermore, the addition of UPF1/(IGF2BP3/IMP3) substantially improved risk reclassification, yielding a continuous NRI of 1.097 (95% CI: 0.795–1.454, *P* < 0.001) and an IDI of 0.078 (95% CI: 0.051–0.115, *P* < 0.001). These findings indicate that the pre-procedural UPF1/(IGF2BP3/IMP3) ratio provides potential exploratory utility for longitudinal post-PCI risk stratification rather than serving as a concurrent diagnostic test.

## 4 Discussion

In the present study, we determined the correlation between RNA-binding protein IGF2BP3/IMP3, UPF1 and the development of ISR in patients after PCI. Our current study indicated that IGF2BP3/IMP3 and VEGF were decreased in ISR patients and the expression of both was positively correlated, while UPF1 was elevated in ISR patients and was inversely correlated with VEGF. An elevated baseline UPF1/IGF2BP3/IMP3 ratio is significantly associated with subsequent ISR after PCI and exhibits strong predictive utility for identifying patients at risk of ISR, although these observational data reflect clinical associations rather than direct pathophysiological causation.

Recent studies have continued to investigate circulating biomarkers for ISR risk assessment. A 2025 systematic review and meta-analysis reported an association between elevated pre-PCI C-reactive protein (CRP) levels and subsequent ISR. Another systematic review and meta-analysis identified the neutrophil-to-lymphocyte ratio (NLR) as a potential predictor of coronary ISR, although heterogeneity across studies remains an important consideration. These findings support continued investigation of circulating biomarkers for ISR risk stratification, while highlighting the need for further validation of their predictive performance and clinical applicability. Against this background, our study explored the associations of baseline IGF2BP3/IMP3, UPF1, and their ratio with subsequent ISR in patients with ACS undergoing PCI (25, 26).

Coronary angioplasty and stent implantation are commonly utilized and effective treatments for ACS, which are generally recognized in clinical practice. However, angioplasty or stent implantation often contributes to in-stent restenosis, hampering the prognosis of patients undergoing PCI (27). DES can promote the proliferation of the original host endothelial cells around the stent, migrate and cover the surface of the stent, and accelerate the process of reendothelialization, which is an important way to reduce the occurrence of ISR and treat ISR. The overall picture of ISR prevention strategies has remained relatively unchanged over the years, despite numerous clinical trials reporting early reduction of in-stent restenosis with next-generation DES (28, 29). The release of stent-coated drugs can inhibit the proliferation of vascular smooth muscle cells and reduce the rate of restenosis after PCI but can also lead to endothelial cell dysfunction, inhibit endothelial cell proliferation and migration, delay stent surface reendothelialization, and increase the risk of ISR. Therefore, it is pertinent to explore certain influencing factors that affect reendothelialization in patients with ISR.

An intriguing finding of our study, now visually represented in Figure 4 is the distinct temporal variation pattern of IGF2BP3/IMP3, VEGF, and UPF1 following PCI. The coordinated rise of IGF2BP3/IMP3 and VEGF, peaking at day 7, chronologically parallels the critical window for endothelial cell proliferation and migration required for stent surface coverage. While previous *in vitro* studies indicated that IGF2BP3/IMP3 promotes VEGF mRNA stabilization, our clinical observations alone cannot confirm a direct regulatory hierarchy in patients, but rather provide correlative evidence in line with this biological hypothesis. Conversely, the sharp decline of UPF1 during this same period parallels these temporal shifts, which may hypothetically reflect changes in mRNA decay activity during the acute vascular repair phase. Rather than establishing a definitive molecular switch governing the vascular healing response, this dynamic interplay reflects an observational clinical phenomenon that warrants mechanistic validation. The pattern of variation of these molecules is consistent with the progression of reendothelialization, and our new index UPF1/(IGF2BP3/IMP3) captures the peak of this divergence, which explains its strong statistical predictive power for ISR.

IGF2BP3/IMP3, a member of the insulin-like growth factor 2 mRNA-binding protein family, which plays a vital role transcript localization, translation and stabilization in post-transcriptional RNA processing (30–35). IGF2BP3/IMP3 is involved in RNA transport and stabilization, thus promoting cell proliferation and migration in tumor progression and metastasis (36, 37). The proliferative and migratory capacity of endothelial cells is crucial in the process of vascular reendothelialization, which plays an indispensable role in ISR (38–40). Endothelial cells express IGF2BP3/IMP3 at relatively modest levels in the absence of injury; whereas IGF2BP3/IMP3 expression level in endothelial cells is significantly elevated after arterial damage. Intriguingly, This variation of IGF2BP3/IMP3 expression after carotid injury is in accordance with the progression of reendothelialization, demonstrating that IGF2BP3/IMP3 may be engaged in the process of reendothelialization after carotid artery injury (41). Cao and colleagues have revealed that high expression of IGF2BP3/IMP3 and VEGF was positively corresponded to the stage of osteosarcoma and microvessel density, which could be a promising indicator of the degree of osteosarcoma malignancy and the prognosis of the patients (42). Furthermore, Yu et al. elucidated that knockdown of IGF2BP3/IMP3 in an endothelial cell line (HUVEC) resulted in the down-regulation of cell proliferation-related proteins (*e.g.*, cell cycle protein B) (43). In conclusion, the current studies have indicated that IGF2BP3/IMP3 can bind VEGF mRNA and regulate its expression. Apart from IGF2BP3/IMP3, UPF1 is also involved in the modulation of VEGF. UPF1 is an ATP-dependent RNA deconjugating enzyme that plays an imperative role in NMD, which is the most typical eukaryotic mRNA monitoring pathway. NMD is typically thought of as an mRNA quality control mechanism that preferentially recognizes and degrades faulty transcripts containing premature termination codons (PTCs). NMD is also engaged in mRNA quantity control through manipulating the number of natural transcripts. In addition to NMD, UPF1 facilitates other mRNA decay pathways mediated by a variety of molecules, such as glucocorticoid receptor, stem-loop binding protein, and regnase−1. Conjecturally, UPF1 is served as RNA-binding proteins to affect the expression of VEGF, participating in the process of reendothelialization and regulating the occurrence of ISR.

Based on existing molecular literature, we hypothesize that the RNA-binding proteins IGF2BP3/IMP3 and UPF1 could participate in post-transcriptional modulation of VEGF expression, thereby potentially contributing to reendothelialization. Importantly, these observed population-level correlations between circulating IGF2BP3/IMP3/UPF1 and VEGF provide observational clinical support for our working hypothesis, but not be construed as definitive proof of direct molecular regulation or direct biochemical interaction *in vivo*. Further mechanistic *in vitro* and animal studies are required to confirm the precise regulatory hierarchy. The results demonstrated that the serum levels of VEGF and IGF2BP3/IMP3 in the ISR group were lower than those in the non-ISR group, while the level of serum UPF1 in the ISR group was higher, indicating that higher baseline circulating levels of IGF2BP3/IMP3 and VEGF are associated with a reduced risk of ISR, whereas elevated UPF1 is an independent risk predictor. These observational findings should not be interpreted as direct evidence that IGF2BP3/IMP3 and VEGF biologically inhibit ISR, or that UPF1 directly promotes neointimal hyperplasia *in vivo*. This study proposed the UPF1/(IGF2BP3/IMP3) ratio as a candidate biomarker signature associated with post-PCI ISR; however, these findings reflect an exploratory association that warrants rigorous prospective and external multicenter validation before any clinical application. Several limitations of this study should be acknowledged. Initially, this is a single-center retrospective study, which may introduce inherent selection bias; thus, large-scale, prospective, multicenter cohorts are warranted to validate our findings. Additionally, potential verification bias must be considered regarding the 6-month angiographic follow-up. Although routine 6-month coronary angiography was recommended to all patients, approximately 16.0% of eligible patients were excluded due to a lack of invasive angiographic follow-up. Consequently, the analyzed cohort may carry a selection bias toward patients who were more compliant or exhibited clinical indications for re-examination, potentially underrepresenting asymptomatic ISR cases. Furthermore, although biomarker measurements utilized for regression and ROC modeling were obtained at baseline (prior to PCI) before the development of angiographic ISR at 6 months, these findings should strictly be interpreted as an independent statistical association rather than definitive causal prediction. Specifically, a clear distinction must be maintained between the observed clinical associations/statistical predictions in our cohort and the underlying cellular mechanisms, which remain working hypotheses. Direct mechanistic and interventional animal studies are warranted to verify whether targeting the (IGF2BP3/IMP3)/UPF1/VEGF pathway can causally alter vascular healing and prevent ISR. Furthermore, multivariate regression mainly adjusted the core systematic clinical risk factors to prevent model overfitting. The potential residual confounding of detailed angiographic and surgical variables need to be further verified in a larger-scale prospective cohort study. Finally, although internal bootstrap validation demonstrated favorable stability, the optimal cutoff and predictive performance of the UPF1/(IGF2BP3/IMP3) ratio were derived within a single-center cohort. Therefore, this ratio should be interpreted strictly as an exploratory predictive indicator rather than an established clinical diagnostic test until validated in prospective, multicenter external cohorts.

## Data Availability

The original contributions presented in the study are included in the article/Supplementary Material, further inquiries can be directed to the corresponding author.
